# Prevalence and Clinical Characteristics of Bacterial Pneumonia in Neurosurgical Emergency Center Patients: A Retrospective Study Spanning 13 Years at a Tertiary Center

**DOI:** 10.3390/microorganisms11081992

**Published:** 2023-08-02

**Authors:** Weijian Yang, Haijun Yao, Caihua Xi, Xiangru Ye, Qifang Chen, Jun Zhang, Jian Yu, Jin Hu

**Affiliations:** 1 Department of Neurosurgery, Huashan Hospital, Shanghai Medical College, Fudan University, Shanghai 200040, China; 2 National Center for Neurological Disorders, Shanghai 200040, China; 3 Shanghai Key Laboratory of Brain Function Restoration and Neural Regeneration, Shanghai 200040, China; 4 Neurosurgical Institute of Fudan University, Shanghai 200040, China; 5 Shanghai Clinical Medical Center of Neurosurgery, Shanghai 200040, China; 6 Department of Neurosurgery and Neurocritical Care, Huashan Hospital, Fudan University, Shanghai 200040, China; 7 Department of Nursing, Huashan Hospital, Shanghai Medical College, Fudan University, Shanghai 200040, China

**Keywords:** brain injury, bacterial pneumonia, resistance rate, antimicrobial agent

## Abstract

Patients with brain injuries are at a heightened susceptibility to bacterial pneumonia, and the timely initiation of empiric antibiotic treatment has been shown to substantially reduce mortality rates. Nevertheless, there is a need for knowledge regarding the resistance and prevalence of pulmonary bacterial infections in this patient population. To address this gap, a retrospective study was conducted at a neurosurgical emergency center, focusing on patients with brain injuries. Among the entire patient population, a total of 739 individuals (18.23%) were identified as having bacterial pneumonia, consisting of 1489 strains of Gram-negative bacteria and 205 strains of Gram-positive bacteria. The resistance of *Klebsiella pneumoniae* to imipenem exhibited a significant increase, rising from 21.74% in 2009 to 96.67% in 2018, and subsequently reaching 48.47% in 2021. *Acinetobacter baumannii* displayed resistance rates exceeding 80.0% against multiple antibiotics. The resistance profile of *Pseudomonas aeruginosa* was relatively low. The proportion of *Staphylococcus aureus* reached its peak at 18.70% in 2016, but experienced a decline to 7.83% in 2021. The abundance of Gram-negative bacteria exceeded that of Gram-positive bacteria by a factor of 5.96. *Klebsiella pneumoniae*, *Acinetobacter baumannii*, and *Staphylococcus aureus* are prominent pathogens characterized by limited antibiotic choices and scarce treatment alternatives for the isolated strains.

## 1. Introduction

Patients with subarachnoid hemorrhage (SAH), stroke, intracerebral hemorrhage (ICH), or traumatic brain injury (TBI) are at an elevated risk of contracting nosocomial infections, specifically pneumonia [1]. Previous cohorts have characterized the epidemiology of infections in brain injury and identified a respiratory source in frequencies as high as 94% of cases [2]. An early initiation of empiric antibiotic therapy helps reduce morbidity, mortality, and medical expenses [3]. Antimicrobial resistance (AMR) poses a growing threat to public health [4], resulting in 3.57 million deaths worldwide in 2019 [5]. Consequently, antibiotic choices become more difficult as AMR becomes more prevalent [6].

It was noted that AMR profiles varied significantly across regions, hospitals, and time periods [7]. To develop an effective empirical therapy for bacterial infections, it is imperative to possess a comprehensive comprehension of the microorganism’s potential and the corresponding susceptibility patterns. As recommended by the Infection Disease Society of America and the American Thoracic Society, all hospitals should regularly generate and distribute local antibiograms [8]. The China Antimicrobial Surveillance Network (CHINET) and China Antimicrobial Resistance Surveillance System (CARSS) are two well-known surveillance networks for bacterial resistance in mainland China [9]. Nevertheless, the microbiological findings commonly lack pertinent epidemiological or clinical information. Furthermore, there is a scarcity of published data regarding the trends of AMR among patients in neurosurgical emergency centers.

Therefore, a retrospective study was conducted to characterize the prevalence and clinical characteristics of bacteria cultured from lower respiratory tract among patients in a neurosurgical emergency center between January 2009 and December 2021.

## 2. Materials and Methods

### 2.1. Study Design and Settings

A retrospective study was conducted to investigate trends in AMR among patients with brain injury who were diagnosed with bacterial pneumonia and had bacterial resistance reports over a thirteen-year period, from January 2009 to December 2021, at the neurosurgical emergency center of Huashan Hospital, Fudan University, China. As the National Center for Neurological Disorders and one of the oldest neurosurgical departments in China, Huashan Hospital has established the Shanghai Emergency Center of Neurosurgery [10]. The Ethics Committee of the Evaluation of Biomedical Research Projects of Huashan Hospital granted approval for this study (Number: 2022-877). As a retrospective and non-interventional clinical research, the requirement for informed consent was waived.

The diagnosis of bacterial pneumonia necessitates the acquisition of positive cultures from endotracheal aspirates or bronchoscopic sampling techniques [11]. In addition, the presence of abnormal radiological findings indicating new or progressed pulmonary infiltrate(s) and clinical signs of infection, including the onset of fever (≥38 °C), increased sputum production and/or a change in sputum color to a more purulent state, peripheral leukocytosis, and decreased oxygenation or the requirement of oxygen supplement therapy, are required criteria [12].

### 2.2. Bacterial Isolates and Susceptibility Testing

The bacterial isolates and AMR data were obtained from the hospital information system. The disk diffusion method, in accordance with the Clinical and Laboratory Standards Institute (CLSI) criteria, was utilized to determine the antibiotic susceptibilities of clinical isolates. The methods for antimicrobial susceptibility testing and quality control were previously documented by other researchers [13]. During the same in-patient stay, we retained the first isolates of duplicate test results from the same patient.

### 2.3. Statistical Analysis

The statistical analysis of nominal variables was conducted using the Chi-square test, while the analysis of continuous variables was performed using the Student’s *t*-test. A significance level of *p* < 0.05 was deemed appropriate to indicate statistical significance. All calculations were executed using SPSS (version 20.0). Sankey diagram was performed using the OmicStudio tools at https://www.omicstudio.cn/tool (accessed on 25 July 2023).

## 3. Results

### 3.1. Demographics and Epidemiology

This study included a total of 4054 patients with brain injury and cerebral hemorrhage from January 2009 to December 2021. Among them, no bacteria were cultured from samples in the 3315 (81.77%) patients, whose average age was 51.99 ± 16.40. The average length of hospital stay was much longer in patients cultured with bacteria than in patients without bacteria (22.79 ± 12.17 to 14.04 ± 8.70, *p* < 0.0001). In the cohort of patients without bacteria, 2425 (73.15%) were male and 890 (26.85%) were female. Furthermore, there were 2499 (75.38%) patients who were diagnosed with TBI, while 816 (24.62%) were diagnosed with ICH.

Out of the total number of patients, 739 (18.23%) were diagnosed with bacterial pneumonia and presented with cultured isolates. The mean age of these patients was 50.38 ± 13.30. Among the cases, 592 (80.11%) were male and 147 (19.89%) were female. The dataset comprised of 479 (64.82%) patients with TBI and 260 (35.18%) with ICH. The duration between admission and isolate detection was 6.82 ± 4.44 days. The mortality rate was 3.65%. The data are presented in Table 1.

### 3.2. Trends in Bacterial Isolates

**Figure 1:**
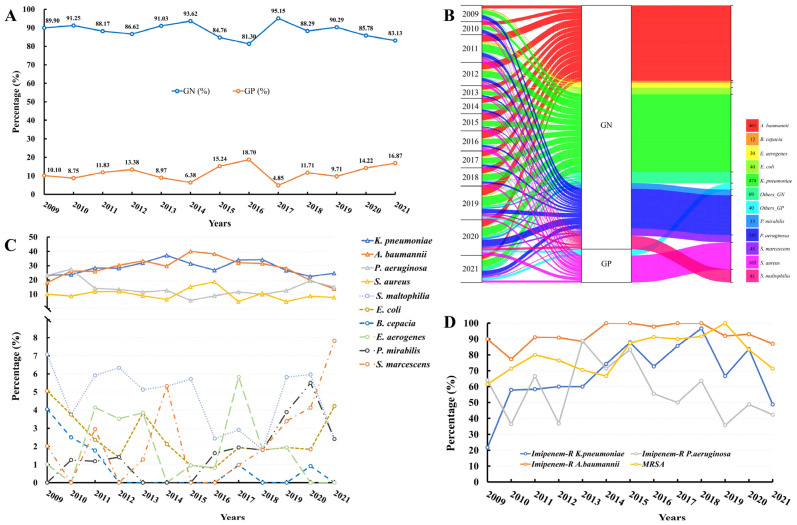
Bacterial isolates and their trends from 2009 to 2021. (**A**). Trends of Gram-negative and Gram-positive bacteria from 2009 to 2021. (**B**). Bacterial isolates from 2009 to 2021. (**C**). Trends of top ten isolates from 2009 to 2021. (**D**). Rates of Imipenem-resistant *K. pneumoniae*, *A. baumannii*, *P. aeruginosa* and MRSA from 2009 to 2021. GN, Gram-negative; GP, Gram-positive; *A. baumannii*, *Acinetobacter baumannii*; *B. cepacian*, *Burkholderia cepacian*; *E. aerogenes*, *Enterobacter aerogenes*; *E. coli*, *Escherichia coli*; *K. pneumoniae*, *Klebsiella pneumoniae*; *P. mirabilis*, *Proteus mirabilis*; *P. aeruginosa*, *Pseudomonsa aeruginosa*; *S. marcescens*, *Serratia marcescens*; *S. aureus*, *Staphylococcus aureus*; *S. maltophilia*, *Stenotrophomonas maltophilia*. Imipenem-R, Imipenem-resistant; MRSA, methicillin-resistant *S. aureus*.

Between 2009 and 2021, the prevalence of Gram-negative (GN) bacteria surpassed that of Gram-positive (GP) bacteria, with a peak of 95.15% in 2017 (Figure 1A). Between 2009 and 2021, the proportion of GN bacteria ranged from 81.30% to 95.15% (Figure 1A). Conversely, the proportion of GP bacteria exhibited an upward trajectory, reaching 16.87% in 2021 (Figure 1A).

Between 2009 and 2021, a comprehensive analysis of GN bacteria yielded 37 distinct species, comprising a total of 1489 strains. Among these, *Klebsiella pneumoniae* (*K. pneumoniae*) accounted for 474 strains, *Acinetobacter baumannii* (*A. baumannii*) for 461 strains, *Pseudomonsa aeruginosa* (*P. aeruginosa*) for 242 strains, and *Stenotrophomonas maltophilia* (*S. maltophilia*) for 81 strains (Figure 1B). Additionally, a total of 205 strains of GP bacteria were isolated, encompassing 15 species, with *Staphylococcus aureus* (*S. aureus*) being the most prevalent, accounting for 165 strains (Figure 1B).

The top 10 bacterial isolates from 2009 to 2021 were subjected to further analysis. The results indicate that the proportion of *K. pneumoniae* ranged from 22.5% to 37.2%. Similarly, *A. baumannii* experienced a decrease from its peak of 40.0% in 2015 to 13.9% in 2021. *P. aeruginosa* demonstrated a V-shaped trend from 2009 to 2021, with an overall downward trend from 2009 to 2015, reaching its lowest level of 5.7% in 2015, and an overall upward trend from 2015 to 2021, reaching 15.1% in 2021. Finally, *S. aureus* reached its highest level of 18.7% in 2016. From 2016 to 2021, *Escherichia coli* (*E. coli*) exhibited a W-shaped trend, with a proportion of 4.2% in 2021. Conversely, *Serratia marcescens* (*S. marcescens*) demonstrated a consistent upward trend from 2017 to 2021, with a proportion of 7.8% in 2021. The data are presented in Figure 1C.

### 3.3. AMR Patterns of Top Four Bacteria

#### 3.3.1. *Klebsiella pneumoniae*

**Figure 2:**
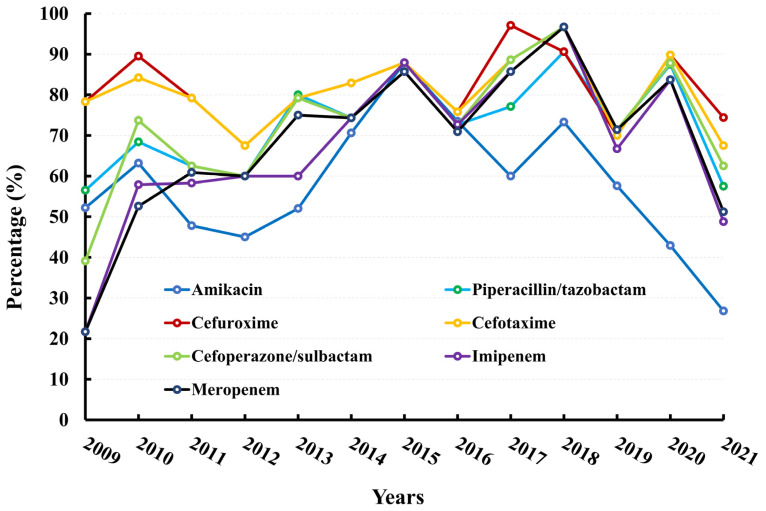
Resistance rates (%) of *K. pneumoniae* to antimicrobial agents.

The AMR rates of cefuroxime and cefotaxime exhibited similar trends. Over the course of the past 13 years, the average resistance rate of cefuroxime was 81.69%, with a notable increase from 2012 to 2017 and a peak of 97.14% in 2017. Similarly, the average resistance rate of cefotaxime over the same period was 80.56%, with a general upward trend from 2012 to 2018 and a peak of 96.67%. These findings suggest that approximately 80% of *K. pneumoniae* strains produce extended-spectrum β-lactamase (ESBL). The AMR rates of piperacillin–tazobactam and cefoperazone-sulbactam exhibit a similar trend to that of imipenem or meropenem from 2009 to 2021. The level of resistance to amikacin rose from 52.17% in 2009 to 87.88% in 2015, but subsequently demonstrated a fluctuating downward trend, reaching 26.83% in 2021. It is noteworthy that the AMR rates of amikacin displayed this fluctuation. The data are presented in Figure 2 and Supplementary Table S1.

A notable observation is the significant increase in resistance to imipenem, rising from 21.74% in 2009 to 96.67% in 2018, followed by a fluctuating downward trend to 48.47% in 2021 (Figure 1D). The AMR rates of meropenem and imipenem exhibited a similar pattern. The data are presented in Figure 2 and Supplementary Table S1.

#### 3.3.2. *Acinetobacter baumanni*

**Figure 3:**
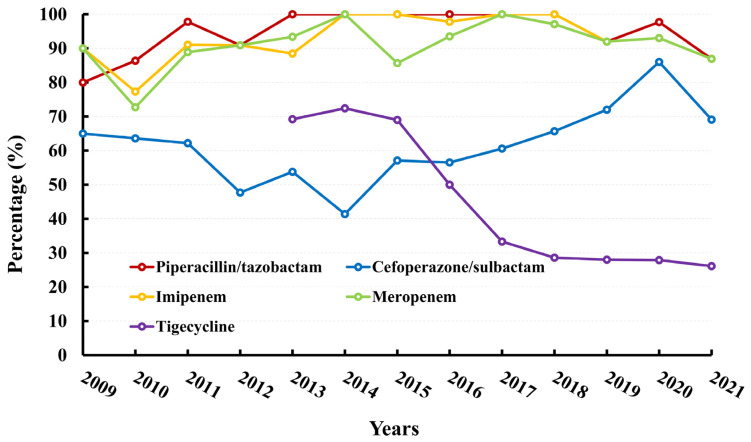
Resistance rates (%) of *A. baumannii* to antimicrobial agents.

The AMR rate of *A. baumannii* to multiple antibiotics has remained consistently high, with an average of approximately 90% (Supplementary Table S2). This trend is exemplified by the AMR rates of imipenem and meropenem, which have remained at 92.89% and 91.08%, respectively, over the past 13 years. Notably, the AMR rates of cefoperazone-sulbactam have increased from 47.73% in 2012 to 86.05% in 2020, while the AMR rates of tigecycline have decreased from 69.23% to 26.09%. The data are presented in Figure 1D and Figure 3 and Supplementary Table S2. It is worth noting that no isolate was found to be resistant to polymyxin (Supplementary Table S2).

#### 3.3.3. *Pseudomonas aeruginosa*

**Figure 4:**
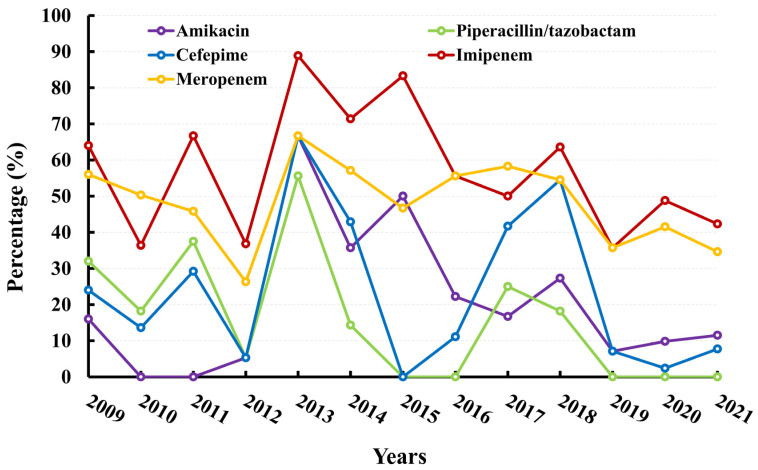
Resistance rates (%) of *P. aeruginosa* to antimicrobial agents.

In general, *P. aeruginosa* demonstrated a relatively moderate degree of resistance, as evidenced by the decline in AMR rates for imipenem and meropenem from 64.00% in 2009 to 42.31% in 2021 and from 56% in 2009 to 34.62% in 2021, respectively. Additionally, the AMR rates for piperacillin–tazobactam decreased from 32% to 0%. The data are presented in Figure 4 and Supplementary Table S3.

#### 3.3.4. *Staphylococcus aureus*

**Figure 5:**
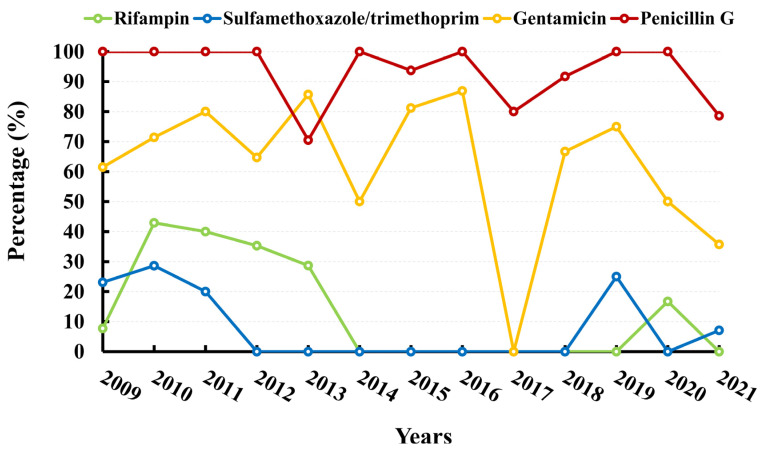
Resistance rates (%) of *S. aureus* to antimicrobial agents.

The present study reports on the incidence of methicillin-resistant *S. aureus* (MRSA) over a period of seven years, from 2014 to 2021. The results indicate a rising trend in MRSA incidence, with a peak of 100% in 2019, followed by a subsequent decline to 71.43% in 2021. Notably, no isolates were found to be resistant to vancomycin, linezolid, or teicoplanin. The AMR rate to rifampin exhibited significant fluctuations, ranging from 42.86% in 2010 to 0.00% in 2021. Similarly, the AMR rates to sulfamethoxazole–trimethoprim and gentamicin decreased from 23.08% to 7.14% and from 61.54% to 35.71%, respectively. The data are presented in Figure 1D and Figure 5 and Supplementary Table S4.

## 4. Discussion

Individuals who have sustained injuries to the central nervous system (CNS) are at an increased risk for pneumonia, which can be further exacerbated by various factors such as prolonged bed rest, dysphagia, cognitive impairment, or mechanical ventilation due to weakened respiratory muscles [14]. Pneumonia is a common complication that affects up to 60% of those with severe brain injuries, largely due to prolonged periods of prone positioning and the attendant risk of aspiration of gastric contents [14]. Adherence to European treatment guidelines necessitates the prompt and suitable administration of empirical antibiotics for the effective management of hospital-acquired pneumonia (HAP) and ventilator-associated pneumonia (VAP) [15]. The injudicious use of antibiotics in clinical settings has been identified as a causative agent in the emergence and proliferation of antibiotic resistance.

Numerous articles have documented the resistance and patterns of pulmonary bacterial infection; however, their applicability to patients with brain injury is limited. In light of this, the present study aimed to investigate the resistance and patterns of bacteria cultured from the lower respiratory tract obtained from patients with severe neurological conditions. The study sample comprised 4054 patients with brain injury, of whom 18.23% were diagnosed with bacterial pneumonia and had AMR reports. The study found that the mean age of individuals who tested with bacteria was 50.38 years, and those who tested without bacteria had a shorter mean duration of hospitalization (*p* < 0.0001). The average time between the first AMR report and admission was 6.82 days. The prevalence of GN bacteria in China is notably higher than that of GP bacteria [13]. We identified 37 species of GN bacteria and 15 species of GP bacteria isolated between 2009 and 2021, with the former being 5.96 times more prevalent than the latter. The present investigation revealed that *K. pneumoniae*, *A. baumannii*, *P. aeruginosa*, and *S. aureus* were the four highest-ranking isolates, representing 27.98%, 27.21%, 14.29%, and 9.74%, respectively. These findings are in agreement with the data reported by the CHINET [13]. In Europe, the most frequently isolated microorganisms in ICU-acquired pneumonia episodes were *Klebsiella* spp. (18.8%), followed by *S. aureus* (18.7%), *P. aeruginosa* (16.1%), *Escherichia coli* (13.3%), *Enterobacter* spp. (9.4%) [16]. The reasons for the differences between the data we report and those in Europe may be as follows: Our study focused on patients with pneumonia in the NICU, whereas the European data were based on ICU patients and the European data were derived from multiple centers. *K. pneumoniae* is a commonly encountered pathogen, with carbapenem-resistant *K. pneumoniae* (CRKP) exhibiting a substantial increase worldwide in recent decades, thereby posing a pressing public health threat [17]. In China, the incidence of imipenem-resistant *K. pneumoniae* has been steadily rising since 2005, reaching 25.0% in 2018 [18]. In Europe, the percentage of CRKP remained stable from 2017 to 2018, and increased by +8% from 2018 to 2019. In 2020, the percentage increased by a further +31%, and by another +20% in 2021 [19]. However, despite the widespread implementation of AMR surveillance programs, these initiatives typically have limited temporal scope and do not account for specific disease identification [20,21,22]. In our investigation, the prevalence of *K. pneumoniae* isolates demonstrated a fluctuation ranging from 20% to 40%. The limited availability of antibiotics and treatment options for these isolates is a concern. Over the course of the past 13 years, the average AMR rates for cefuroxime and cefotaxime were 81.69% and 80.56%, respectively. This data suggests that approximately 80% of *K. pneumoniae* strains exhibit ESBL production. Notably, resistance to imipenem increased from 21.74% in 2009 to 96.67% in 2018, but decreased to 48.47% in 2021. Our findings indicate that the incidence of CRKP isolates in patients with brain injury was significantly higher than that of the CHINET. Moreover, our investigation unveiled substantial temporal variations, underscoring the significance of conducting extended antibiotic resistance monitoring. Notably, certain antimicrobial agents, such as carbapenems, warrant particular attention in this regard.

*A. baumannii*, a multidrug-resistant nosocomial pathogen, is responsible for a significant number of hospital-acquired infections. In intensive care units, VAP caused by multiple drug-resistant *A. baumannii* has been reported to have an 84.3% mortality rate [23]. CHINET monitoring data indicates that the prevalence of carbapenem-resistant *A. baumannii* (CRAB) has increased from 31.0% in 2005 to 71.5% in 2021 [24]. In Europe, the ratio of CRAB was 82.3% in 2019 [16]. Our findings reveal a decrease in *A. baumannii* prevalence to 13.86% in 2021. The AMR rates of *A. baumannii* to multiple antibiotics, such as amikacin, cefepime, ceftazidime, ciprofloxacin, piperacillin/tazobactam, imipenem, and meropenem, exceeded 80.0%. This finding is consistent with a report encompassing 13 regions or countries [25].

Ceftazidime–avibactam and cefiderocol are considered to be the optimal antimicrobial therapies for the treatment of *A. baumannii* infections caused by carbapenem-resistant GN pathogens. A study conducted in 2023 revealed a tigecycline resistance rate of 21.1% from 2014 to 2019 [26]. In contrast, our investigation demonstrated an AMR rate of 69.23% in 2013, which gradually declined to 26.09% in 2021. Notably, no isolate exhibited resistance to polymyxin. Furthermore, the prevalence of tigecycline-resistant *A. baumannii* varied across different countries or regions [27]. Hence, it is imperative to consistently monitor the resistance profiles of *A. baumannii* in diverse geographical areas.

The swift mutations and adaptations that facilitate the acquisition of antibiotic resistance by *P. aeruginosa* infections render them a worldwide health concern, rather than a localized threat [28]. CHINET data reveals that *P. aeruginosa* accounted for 7.96% of nosocomial infections, following *E. coli*, *K. pneumoniae*, and *S. aureus* [29]. In Europe, *P. aeruginosa* accounted for 16.1% from ICU-acquired pneumonia in 2019 [16]. In our investigation, *P. aeruginosa* ranked third among sputum culture bacteria in the neurosurgery emergency center, representing 15.06% of cases in 2021. The findings of a study conducted in Zhejiang, China from 2015 to 2017 indicated that a significant proportion of *P. aeruginosa* exhibited resistance to imipenem and meropenem, with rates of 37.26% and 29%, respectively [30]. In Europe, the ratio of *P. aeruginosa* exhibited resistance to Carbapenem was 25.5% in 2019 [16]. In contrast, our study conducted in 2021 revealed a comparatively low resistance profile of *P. aeruginosa*, with resistance rates to pipercillin–tazobactam, cefepime, amikacin, and ceftazidime at 0.00%, 7.69%, 11.54%, and 11.54%, respectively. However, the resistance rate for imipenem was found to be 42.31% in 2021. The aforementioned dissimilarities suggest that the bacteria present in various medical facilities exhibit distinct resistance profiles, and the frequency of resistance varies significantly.

MRSA, a pathogen responsible for nosocomial infections, has been found to cause increased hospitalization expenses and prolonged stays [31]. In China, the prevalence of MRSA was reported to be 69% in 2005, but has since shown a steady decline [32]. However, recent data from CHINET indicates that over 30% of *S. aureus* isolates were MRSA in 2021 [33]. In Europe, *S. aureus* decreased from 18.4% to 15.8% during the period 2017–2021. Nevertheless, MRSA remains an important pathogen with percentages remaining high in several countries [19]. Our study revealed that *S. aureus* was the fourth most common bacteria found in sputum isolates of patients with brain injury. The proportion of *S. aureus* peaked at 18.70% in 2016 and subsequently decreased to 7.83% in 2021, with MRSA proportions showing similar fluctuations. In 2019, the prevalence of MRSA reached 100%, subsequently exhibiting a declining trajectory and attaining a rate of 71.43% in 2021.

According to CHINET, staphylococcal strains did not exhibit vancomycin resistance, although a small percentage of methicillin-resistant coagulase-negative staphylococci strains were resistant to linezolid [34]. Another study found that MRSA was completely susceptible to vancomycin and teicoplanin, but a few linezolid-resistant strains were identified in 2016 [35]. Our study supports these results, as no instances of resistance to vancomycin, linezolid, or teicoplanin were observed in the staphylococcal strains analyzed.

The emergence of multidrug-resistant and super-resistant bacteria has become a major concern for governments worldwide with regard to bacterial resistance. The main drivers of antimicrobial resistance include the misuse and overuse of antimicrobials; poor infection and disease prevention and control in health-care facilities; lack of awareness and knowledge; and lack of enforcement of legislation. As a response, the National Health Commission of the People’s Republic of China published a theme in 2020 that focused on the management of the clinical application of antibiotics [26]. In recent years, our hospital has taken extremely strict measures on the use of antibiotics and launched an intelligent infection management system. The system can not only assess the infection of patients by actively capturing their medical data, but also warn of the outbreak of infection. Our surveillance showed no clusters of infection and revealed a decreasing trend in Imipenem-R *K. pneumoniae*, Imipenem-R *A. baumannii*, Imipenem-R *P. aeruginosa*, and MRSA over the past three years. Consequently, it is imperative to closely monitor the resistance rate in medical institutions.

The present study was constrained by specific limitations, such as its retrospective design and limited sample size, which were confined to a solitary tertiary care hospital. As a result, the generalizability of the findings to the wider population may be limited. Nevertheless, despite these limitations, the retrospective study on bacterial pneumonia in patients with brain injury yielded significant insights into the shifts and patterns in AMR.

## 5. Conclusions

This retrospective study spanning 13 years aimed to examine the resistance patterns and trends of bacteria in lower respiratory tract isolates obtained from patients with brain injury during the period of January 2009 to December 2021. The susceptibility profiles of *K. pneumoniae*, *A. baumannii*, *P. aeruginosa*, and *S. aureus* can provide valuable guidance to neurosurgeons in making informed decisions regarding antibiotic therapy, thereby mitigating the risk of irrational antibiotic use. The findings underscore the importance of selecting appropriate antibiotic treatments based on the antibiogram outcomes. The results of this investigation have practical implications for individuals suffering from bacterial pneumonia and brain injuries, as they may experience a decrease in hospitalization duration and related expenses.

## Figures and Tables

**Table 1:** Demographic and clinical characteristics of brain injury patients with bacterial pneumonia.

Parameters	Total	TBI	ICH	*p*
Cases	739	479	260	-
Age	50.4 ± 13.3	48.9 ± 13.6	53.1 ± 12.4	<0.0001
Sex, male:female	592:147	390:89	202:58	0.2
GCS	8.2 ± 3.3	8.3 ± 3.3	8.2 ± 3.3	0.7
Interval days	6.8 ± 4.4	6.9 ± 4.3	6.6 ± 4.7	0.3
Days in hospital	22.8 ± 12.2	22.3 ± 10.8	23.8 ± 14.4	0.1
Death	27 (3.6%)	17 (3.5%)	10 (3.8%)	-

GCS: Glasgow Coma Scale, Interval days: interval days between infection and the initial injury, TBI: traumatic brain injury, ICH: intracerebral hemorrhage.

## Data Availability

The raw data supporting the conclusions of this article will be made available by the authors, without undue reservation.

## Supplementary Materials

The following supporting information can be downloaded at: https://www.mdpi.com/article/10.3390/microorganisms11081992/s1, Table S1: Resistance rates (%) of *Klebsiella pneumoniae* to antimicrobial agents; Table S2: Resistance rates (%) of *Acinetobacter baumannii* to antimicrobial agents; Table S3: Resistance rates (%) of *Pseudomonas aeruginosa* to antimicrobial agents; Table S4: Resistance rates (%) of *Staphylococcus aureus* to antimicrobial agents.

## References

[1] Erfani Z., Mamaghani H.J., Rawling J.A., Eajazi A., Deever D., Mirmoeeni S., Jafari A.A., Seifi A. (2022). Pneumonia in Nervous System Injuries: An Analytic Review of Literature and Recommendations. Cureus.

[2] Caceres E., Olivella J.C., Yanez M., Viñan E., Estupiñan L., Boada N., Martin-Loeches I., Reyes L.F. (2023). Risk factors and outcomes of lower respiratory tract infections after traumatic brain injury: A retrospective observational study. Front. Med..

[3] Roquilly A., Feuillet F., Seguin P., Lasocki S., Cinotti R., Launey Y., Thioliere L., Le Floch R., Mahe P.J., Nesseler N. (2016). Empiric antimicrobial therapy for ventilator-associated pneumonia after brain injury. Eur. Respir. J..

[4] Tacconelli E., Sifakis F., Harbarth S., Schrijver R., van Mourik M., Voss A., Sharland M., Rajendran N.B., Rodríguez-Baño J., Bielicki J. (2018). Surveillance for control of antimicrobial resistance. Lancet Infect. Dis..

[5] Marutescu L.G. (2023). Current and Future Flow Cytometry Applications Contributing to Antimicrobial Resistance Control. Microorganisms.

[6] Chaibi K., Pean D.P.G., Dortet L., Zahar J.R., Pilmis B. (2022). Empiric Treatment in HAP/VAP: “Don’t You Want to Take a Leap of Faith?”. Antibiotics.

[7] Abdalla J.S., Albarrak M., Alhasawi A., Al-Musawi T., Alraddadi B.M., Wali W.A., Elhoufi A., Habashy N., Hassanien A.M., Kurdi A. (2023). Narrative Review of the Epidemiology of Hospital-Acquired Pneumonia and Ventilator-Associated Pneumonia in Gulf Cooperation Council Countries. Infect. Dis. Ther..

[8] Kalil A.C., Metersky M.L., Klompas M., Muscedere J., Sweeney D.A., Palmer L.B., Napolitano L.M., O’Grady N.P., Bartlett J.G., Carratala J. (2016). Management of Adults with Hospital-acquired and Ventilator-associated Pneumonia: 2016 Clinical Practice Guidelines by the Infectious Diseases Society of America and the American Thoracic Society. Clin. Infect. Dis..

[9] Hu F., Zhu D., Wang F., Wang M. (2018). Current Status and Trends of Antibacterial Resistance in China. Clin. Infect. Dis..

[10] Wang C., Mao Y., Zhu J.H., Zhou L.F. (2008). The Department of Neurosurgery at Shanghai Huashan Hospital. Neurosurgery.

[11] Fernando S.M., Tran A., Cheng W., Klompas M., Kyeremanteng K., Mehta S., English S., Muscedere J., Cook D.J., Torres A. (2020). Diagnosis of ventilator-associated pneumonia in critically ill adult patients-a systematic review and meta-analysis. Intensive Care Med..

[12] Jitmuang A., Puttinad S., Hemvimol S., Pansasiri S., Horthongkham N. (2022). A multiplex pneumonia panel for diagnosis of hospital-acquired and ventilator-associated pneumonia in the era of emerging antimicrobial resistance. Front. Cell. Infect. Microbiol..

[13] Hu F., Guo Y., Yang Y., Zheng Y., Wu S., Jiang X., Zhu D., Wang F. (2019). Resistance reported from China antimicrobial surveillance network (CHINET) in 2018. Eur. J. Clin. Microbiol. Infect. Dis..

[14] Georgakopoulou V.E., Gkoufa A., Aravantinou-Fatorou A., Trakas I., Trakas N., Faropoulos K., Paterakis K., Fotakopoulos G. (2023). Lower respiratory tract infections due to multi-drug resistant pathogens in central nervous system injuries (Review). Biomed. Rep..

[15] Torres A., Niederman M.S., Chastre J., Ewig S., Fernandez-Vandellos P., Hanberger H., Kollef M., Bassi G.L., Luna C.M., Martin-Loeches I. (2017). International ERS/ESICM/ESCMID/ALAT guidelines for the management of hospital-acquired pneumonia and ventilator-associated pneumonia: Guidelines for the management of hospital-acquired pneumonia (HAP)/ventilator-associated pneumonia (VAP) of the European Respiratory Society (ERS), European Society of Intensive Care Medicine (ESICM), European Society of Clinical Microbiology and Infectious Diseases (ESCMID) and Asociacion Latinoamericana del Torax (ALAT). Eur. Respir. J..

[16] Healthcare-Associated Infections Acquired in Intensive Care Units—Annual Epidemiological Report for 2019. https://www.ecdc.europa.eu/en/publications-data/healthcare-associated-infections-intensive-care-units-2019.

[17] Aldali H.J., Khan A., Alshehri A.A., Aldali J.A., Meo S.A., Hindi A., Elsokkary E.M. (2023). Hospital-Acquired Infections Caused by Carbapenem-Resistant Enterobacteriaceae: An Observational Study. Microorganisms.

[18] Hu Y., Liu C., Shen Z., Zhou H., Cao J., Chen S., Lv H., Zhou M., Wang Q., Sun L. (2020). Prevalence, risk factors and molecular epidemiology of carbapenem-resistant Klebsiella pneumoniae in patients from Zhejiang, China, 2008–2018. Emerg. Microbes Infect..

[19] Antimicrobial Resistance in the EU/EEA (EARS-Net)—Annual Epidemio-Logical Report for 2021. https://www.ecdc.europa.eu/en/publications-data/surveillance-antimicrobial-resistance-europe-2021.

[20] Coque T.M., Canton R., Perez-Cobas A.E., Fernández-De-Bobadilla M.D., Baquero F. (2023). Antimicrobial Resistance in the Global Health Network: Known Unknowns and Challenges for Efficient Responses in the 21st Century. Microorganisms.

[21] Collineau L., Bourely C., Rousset L., Berger-Carbonne A., Ploy M.-C., Pulcini C., Colomb-Cotinat M. (2023). Towards One Health surveillance of antibiotic resistance: Characterisation and mapping of existing programmes in humans, animals, food and the environment in France, 2021. Eurosurveillance.

[22] Iera J., Seghieri C., Tavoschi L., Isonne C., Baccolini V., Petrone D., Agodi A., Barchitta M., Arnoldo L., Creti R. (2023). Early Warning Systems for Emerging Profiles of Antimicrobial Resistance in Italy: A National Survey. Int. J. Environ. Res. Public Health.

[23] Shadan A., Pathak A., Ma Y., Pathania R., Singh R.P. (2023). Deciphering the virulence factors, regulation, and immune response to Acinetobacter baumannii infection. Front. Cell. Infect. Microbiol..

[24] Zeng M., Xia J., Zong Z., Shi Y., Ni Y., Hu F., Chen Y., Zhuo C., Hu B., Lv X. (2023). Guidelines for the diagnosis, treatment, prevention and control of infections caused by carbapenem-resistant gram-negative bacilli. J. Microbiol. Immunol. Infect..

[25] Lee Y.L., Ko W.C., Hsueh P.R. (2023). Geographic patterns of Acinetobacter baumannii and carbapenem resistance in the Asia-Pacific Region: Results from the Antimicrobial Testing Leadership and Surveillance (ATLAS) program, 2012–2019. Int. J. Infect. Dis..

[26] Zhou H., Sun X., Lyu S., Yu X., Li R., Wang H., An Z. (2023). Evaluation of Tigecycline Utilization and Trends in Antibacterial Resistance from 2018 to 2021 in a Comprehensive Teaching Hospital in China. Infect. Drug Resist..

[27] Hafiz T.A., Alghamdi S.S., Mubaraki M.A., Alghamdi S.S., Alothaybi A., Aldawood E., Alotaibi F. (2023). A two-year retrospective study of multidrug-resistant Acinetobacter baumannii respiratory infections in critically Ill patients: Clinical and microbiological findings. J. Infect. Public Health.

[28] Losito A.R., Raffaelli F., Del G.P., Tumbarello M. (2022). New Drugs for the Treatment of Pseudomonas aeruginosa Infections with Limited Treatment Options: A Narrative Review. Antibiotics.

[29] Qin S., Xiao W., Zhou C., Pu Q., Deng X., Lan L., Liang H., Song X., Wu M. (2022). Pseudomonas aeruginosa: Pathogenesis, virulence factors, antibiotic resistance, interaction with host, technology advances and emerging therapeutics. Signal Transduct. Target. Ther..

[30] Hu Y.-Y., Cao J.-M., Yang Q., Chen S., Lv H.-Y., Zhou H.-W., Wu Z., Zhang R. (2019). Risk Factors for Carbapenem-Resistant Pseudomonas aeruginosa, Zhejiang Province, China. Emerg. Infect. Dis..

[31] Popovich K.J., Aureden K., Ham D.C., Harris A.D., Hessels A.J., Huang S.S., Maragakis L.L., Milstone A.M., Moody J., Yokoe D. (2023). SHEA/IDSA/APIC Practice Recommendation: Strategies to prevent methicillin-resistant Staphylococcus aureus transmission and infection in acute-care hospitals: 2022 Update. Infect. Control Hosp. Epidemiol..

[32] Wang H. (2022). Current and Future Landscape of the Antimicrobial Resistance of Nosocomial Infections in China. China CDC Wkly..

[33] Yao Z., Wu Y., Xu H., Lei Y., Long W., Li M., Gu Y., Jiang Z., Cao C. (2023). Prevalence and clinical characteristics of methicillin-resistant Staphylococcus aureus infections among dermatology inpatients: A 7-year retrospective study at a tertiary care center in southwest China. Front. Public Health.

[34] Hu F., Yuan L., Yang Y., Xu Y., Huang Y., Hu Y., Ai X., Zhuo C., Su D., Shan B. (2022). A multicenter investigation of 2,773 cases of bloodstream infections based on China antimicrobial surveillance network (CHINET). Front. Cell. Infect. Microbiol..

[35] Huang L., Zhang R., Hu Y., Zhou H., Cao J., Lv H., Chen S., Ding S., Chen G. (2019). Epidemiology and risk factors of methicillin-resistant Staphylococcus aureus and vancomycin-resistant enterococci infections in Zhejiang China from 2015 to 2017. Antimicrob. Resist. Infect. Control.

