# Supplementary material for: Prevalence and Clinical Characteristics of Bacterial Pneumonia in Neurosurgical Emergency Center Patients: A Retrospective Study Spanning 13 Years at a Tertiary Center
Source: Microorganisms. 2023 Aug 2;11(8):1992. doi: 10.3390/microorganisms11081992 (PMC10458519; doi:10.3390/microorganisms11081992)
Supplement: Supplementary file 1 [file microorganisms-11-01992-s001.zip › microorganisms-2503239-supplementary.pdf]

Supplementary Table S1. Resistance rates (%) of *Klebsiella pneumoniae* to antimicrobial agents

Antimicrobial agent	2009	2010	2011	2012	2013	2014	2015	2016	2017	2018	2019	2020	2021
**Amikacin**	52.2	63.2	47.8	45.0	52.0	70.6	87.9	73.5	60.0	73.3	57.6	42.9	26.8
Gentamicin	82.6	78.9	67.4	55.0	76.0	82.9	87.9	75.8	82.9	75.7	65.0	85.7	65.0
**Piperacillin/tazobactam**	56.5	68.4	62.5	60.0	80.0	74.3	87.9	72.7	77.1	90.6	71.4	87.5	57.5
Cefazolin	82.6	83.9	88.5	70.0	80.0	82.9	87.9	75.8	97.1	84.8	70.0	91.8	70.0
**Cefuroxime**	78.3	89.5	79.2	67.5	79.2	82.9	87.9	75.8	97.1	90.6	70.0	89.8	74.4
**Cefotaxime**	78.3	84.2	79.2	67.5	79.2	82.9	87.9	75.8	88.6	96.7	70.0	89.8	67.5
Ceftazidime	78.3	84.2	66.7	55.0	80.0	74.3	87.5	75.8	91.4	96.7	71.4	87.8	63.4
Cefepime	78.3	78.9	60.4	47.4	70.8	77.1	87.9	75.8	91.4	96.7	71.4	89.6	65.0
**Cefoperazone/sulbactam**	39.1	73.7	62.5	60.0	79.2	74.3	87.9	72.7	88.6	96.7	71.4	87.8	62.5
**Imipenem**	21.7	57.9	58.3	60.0	60.0	74.3	87.9	72.7	85.7	96.7	66.7	83.7	48.8
**Meropenem**	21.7	52.6	60.9	60.0	75.0	74.3	85.7	70.9	85.7	96.7	71.4	83.7	51.2
Ciprofloxacin	60.9	84.2	52.1	47.5	48.0	74.3	90.9	72.7	88.6	76.3	76.2	93.9	67.5
Sulfamethoxazole/trimethoprim	78.3	89.5	58.3	37.5	64.0	62.8	84.4	72.7	74.3	78.9	71.4	81.6	48.8

Supplementary Table S2. Resistance rates (%) of *Acinetobacter baumannii* to antimicrobial agents

Antimicrobial agent	2009	2010	2011	2012	2013	2014	2015	2016	2017	2018	2019	2020	2021
Amikacin	90.0	86.4	86.7	84.1	92.3	89.7	92.9	91.3	90.9	91.4	72.0	88.4	82.6
Gentamicin	90.0	95.4	91.1	88.6	96.1	100	95.2	91.3	93.9	91.4	88.0	97.7	86.9
**Piperacillin/tazobactam**	80.0	86.4	97.8	90.9	100	100	100	100	100	97.1	92.0	97.7	86.9
**Cefoperazone/sulbactam**	65.0	63.6	62.2	47.7	53.8	41.4	57.1	56.5	60.6	65.7	72.0	86.0	69.1
Ceftazidime	85.0	86.4	95.6	84.1	100	100	100	100	96.7	100	92.0	97.7	86.9
Cefepime	90.0	86.4	95.6	86.4	95.2	100	100	100	100	91.4	84.0	76.7	52.2
**Imipenem**	90.0	77.3	91.1	90.9	88.5	100	100	97.8	100	100	92.0	93.0	86.9
**Meropenem**	90.0	72.7	88.9	90.9	93.4	100	85.7	93.5	100	97.1	92.0	93.0	86.9
Ciprofloxacin	90.0	86.4	93.3	88.6	100	100	100	100	96.9	100	92.0	97.7	86.9
Sulfamethoxazole/trimethoprim	90.0	81.8	93.3	88.6	92.3	89.7	100	93.8	93.9	91.4	84.0	88.4	78.3
**Tigecycline**	/	/	/	/	69.2	72.4	69.0	50.0	33.3	28.6	28.0	27.9	26.1
Polymyxin	/	/	/	/	/	/	/	/	/	/	0	0	0

"/" indicates the values were not detected.

Supplementary Table S3. Resistance rates (%) of *Pseudomonas aeruginosa* to antimicrobial agents

Antimicrobial agent	2009	2010	2011	2012	2013	2014	2015	2016	2017	2018	2019	2020	2021
**Amikacin**	16.0	0	0	5.3	66.7	35.7	50.0	22.2	16.7	27.3	7.1	9.8	11.5
Gentamicin	32.0	4.5	12.5	5.3	77.8	35.7	50.0	22.2	16.7	27.3	50.0	34.1	30.8
**Piperacillin/tazobactam**	32.0	18.2	37.5	5.3	55.6	14.3	0	0	25.0	18.2	0	0	0
Cefoperazone/sulbactam	28.0	18.2	37.5	5.3	66.7	50.0	50.0	22.2	41.7	27.3	21.4	36.6	30.8
Ceftazidime	24.0	13.6	20.8	5.3	44.4	28.6	16.7	11.1	33.3	54.5	7.1	4.9	11.5
**Cefepime**	24.0	13.6	29.2	5.3	66.7	42.9	0	11.1	41.7	54.5	7.1	2.4	7.7
Aztreonam	48.0	45.4	45.8	26.3	33.3	28.6	0	11.1	25.0	54.5	21.4	4.9	19.2
**Imipenem**	64.0	36.4	66.7	36.8	88.9	71.4	83.3	55.6	50.0	63.6	35.7	48.8	42.3
**Meropenem**	56.0	50.3	45.8	26.3	66.7	57.1	46.7	55.6	58.3	54.5	35.7	41.5	34.6
Ciprofloxacin	44.0	27.3	16.7	15.8	77.8	57.1	50.0	33.3	25.0	45.4	21.4	34.1	42.3
Levofloxacin	/	/	/	/	/	/	/	/	/	9.1	42.9	51.2	57.7

"/" indicates the values were not detected.

Supplementary Table S4. Resistance rates (%) of *Staphylococcus aureus* to antimicrobial agents

Antimicrobial agent	2009	2010	2011	2012	2013	2014	2015	2016	2017	2018	2019	2020	2021
**MRSA***	61.5	71.4	80.0	76.5	70.5	66.7	87.5	91.3	90.0	91.7	100	83.3	71.4
**Vancomycin**	0	0	0	0	0	0	0	0	0	0	0	0	0
**Linezolid**	0	0	0	0	0	0	0	0	0	0	0	0	0
**Teicoplanin**	0	0	0	0	0	0	0	0	0	0	0	0	0
**Rifampin**	7.7	42.9	40.0	35.3	28.7	0	0	0	0	0	0	16.7	0
**Sulfamethoxazole/trimethoprim**	23.1	28.6	20.0	0	0	0	0	0	0	0	25.0	0	7.1
Levofloxacin	61.5	71.4	80.0	58.8	85.7	66.7	87.5	82.6	20.0	83.3	75.0	66.7	64.3
**Gentamicin**	61.5	71.4	80.0	64.7	85.7	50.0	81.2	86.9	0	66.7	75.0	50.0	35.7
Clindamycin	53.8	57.1	70.0	76.5	85.7	83.3	93.7	86.9	20.0	83.3	100	83.3	50.0
Erythromycin	53.8	57.1	70.0	82.3	85.7	83.3	93.7	86.9	20.0	83.3	100	83.3	50.0
**Penicillin G**	100	100	100	100	70.5	100	93.7	100	80.0	91.7	100	100	78.6
Oxacillin	61.5	71.4	80.0	70.6	70.5	66.7	87.5	91.3	90.0	75.0	100	77.8	64.3

*MRSA: methicillin-resistant *Staphylococcus aureus.*
